# REIMAGINING ADRD IN CONTEXT

**DOI:** 10.1093/geroni/igac059.869

**Published:** 2022-12-20

**Authors:** Kristine Ajrouch, Toni Antonucci, Damali Martin

## Abstract

This symposium embraces diversity and discovery to address contextual issues in aging, specifically issues of race and ethnicity in the study of cognitive health and Alzheimer's Disease and Related Dementia (ADRD). Rooks and colleagues compare dementia risk among African American and White men and women in the context of work using the longitudinal Health, Aging, and Body Composition data. They consider the effects of productive activities on dementia risk in gender stratified models, adjusting for socio-demographic and genetic factors. Siddiq and colleagues consider the contexts of migration. Using a multi-method approach, they establish priorities for interventions addressing ADRD risk among older adult immigrants and refugees from Afghanistan and the Middle-East and North Africa (MENA) in California. Sayed also investigates the context of migration, and uses qualitative data (N=31) to identify the psychosocial impact of COVID-19 on cognitive aging in Middle Eastern/Arab Americans immigrants and refugees in Michigan. Finally, Meier and colleagues consider contexts of metal exposure for cognitive decline among Latinos aged 65 and older using the Sacramento Area Latino Study on Aging. In total, this symposium highlights the benefits of reimagining contextual factors that influence ADRD to improve our understanding and the potential to reduce health disparities research in underrepresented racial and ethnic populations.

